# Dietary Influence on Nutritional Epidemiology, Public Health and Our Lifestyle

**DOI:** 10.3390/nu15112555

**Published:** 2023-05-30

**Authors:** Lourdes M. Varela

**Affiliations:** 1 Instituto de Biomedicina de Sevilla, IBiS/Hospital Universitario Virgen del Rocío/CSIC/Universidad de Sevilla, 41013 Sevilla, Spain; lvarela@us.es; 2 Departamento de Fisiología Médica y Biofísica, Facultad de Medicina, Universidad de Sevilla, 41009 Sevilla, Spain

This Special Issue of *Nutrients* “Dietary Influence on Nutritional Epidemiology, Public Health and Our Lifestyle”, includes nine original articles and one systematic review related to the associations between some dietary patterns, lifestyle, and socio-demographic factors, analyzed either separately or in combination, with the risk and management of cardiovascular diseases and mental health problems, such as depression and dementia.

Dietary habits differ from person to person and usually are determined by cultural habits and traditions that determine lifestyles linked to the socio-demographic characteristics associated with ethnicity. It is known that dietary habits, lifestyle, and sociodemographic factors impact human health. While individual foods and nutrients are important, overall dietary patterns are more strongly associated with health. The Mediterranean diet is a compilation of the nutritional and dietary habits of the countries of the Mediterranean Basin. There is strong evidence from epidemiological studies that increasing compliance with the Mediterranean diet is associated with a reduction in total and cause-specific mortality. The systematic review by Grao et al. [1] shows that the Mediterranean diet can improve the quality of high-density lipoproteins (HDL) and prevent HDL dysfunctionality, which is a protective factor against cardiovascular disease. The principal product of the Mediterranean Basin is virgin olive oil, which is obtained from different varieties of olives that confer their individual nutritional and organoleptic properties. Epidemiological studies, long- and short-term intervention studies, and postprandial studies have found associations between a diet rich in virgin olive oil and a decrease in cardiovascular risk factors, stroke incidence, and type 2 diabetes. The study by Vazquez-Madrigal et al. [2] suggests that consuming meals enriched in monounsaturated fatty acids (MUFA) from olive oil can protect against dendritic cells postprandial differentiation and potentially prevent the development and progression of inflammatory and autoimmune diseases.

Nowadays, dementia affects about 50 million people globally and is projected to increase to over 130 million by 2050. When this condition affects people under 65, it is called early onset dementia. Epidemiological studies suggest that aging and obesity have negative effects, among other conditions and disease, on cognitive function. Diet is a key modifiable lifestyle factor that may impact on cognitive function as there is no effective treatment for the improvement of cognition or delay decline. The study by Filippini et al. [3] shows that a high intake of cereals and dairy products is associated with an increased risk of early onset dementia, while the intake of some types of fish, vegetables, fruits, and chocolate alongside moderate coffee consumption appears to be beneficial. The study indicates that an increased adherence to the MIND diet (a hybrid of the Mediterranean and DASH—Dietary Approaches to Stop Hypertension—diets) may decrease early onset dementia risk.

In contrast to the beneficial effects associated with an adherence to the Mediterranean and Mediterranean-DASH Intervention for Neurodegenerative Delay (MIND) diets, the study by Llanaj et al. [4] revealed an unhealthy nutrient-based dietary pattern and unsustainable among Hungarian Roma population, which may contribute to Hungarians being one of the most obese and malnourished nations in Europe. The study indicates the identification of nutrient-rich, affordable, healthy, and sustainable dietary patterns for Hungarian population as a foremost public health priority, while also presenting an opportunity to recognize and address social inequalities in nutrition and health. Another study published in this Special Issue also highlighted the importance of a balanced diet that supplies the nutrients our body needs to be healthy, both physically and mentally. The study by Wang et al. [5] suggests that poor diet quality, measured by the Healthy Eating Index (HEI-2015), is associated with elevated depressive symptoms in US adults. This study highlights the importance of a healthy diet in reducing the risk of depression.

In recent years, and in the current climate crisis, the interest in a sustainable diet/lifestyle has increased. Vegetarians and vegans tend to consume more fruits and vegetables, whole grains, legumes, and nuts, and tend to have a lower body mass index and have a lower risk of diabetes, hypertension, and coronary heart disease compared with meat-eaters. However, there is limited understanding of the underlying molecular mechanisms that could help explain the probable ability of vegetarian dietary patterns to prevent chronic diseases. The study by Miles et al. [6] found modest differences in DNA methylation in vegans and non-vegetarians, suggesting that diet can influence DNA methylation patterns, which could have implications for disease risk and prevention linked to lifestyles.

The diet evolves over time, being influenced by food costs, individual preferences and convictions, cultural traditions, and environmental and geographical aspects. However, the constant truth remains that a healthy diet must help protect against malnutrition, as well as prevent noncommunicable diseases. Dietary nutrients play an important role in maintaining the normal physiological function of the body that is dependent on strict control of its blood glucose levels, among other factors. Nutritional management of blood glucose levels is a strategic target in the control of hyperglycemia, a key feature of diabetes. The study by Yin Bai et al. [7] found that daily total vitamin B6 intake could be a possible predictor of recent glycemic control status among non-pregnant American adults older than 20 years of age, supporting its role as a very important molecule necessary for the health and proper functioning of the human body. Closely related to this study, the work by Yeh et al. [8] evaluated the effect of dehulled adlay consumption on blood pressure in spontaneously hypertensive rats and overweight and obese young adults. Hypertension and hyperglycemia are interrelated and strongly predispose to atherosclerotic disease. This study found that the daily intake of dehulled adlay, a grain rich in phenolic compounds popular in Asian cuisine, had beneficial effects in the management of blood pressure, which was more evident in participants with high basal blood pressure.

Previous studies confirm the importance of a healthy diet. However, the consumption of high-caloric foods, fats, sugar, and salt has increased, and many people do not eat enough fruit, vegetables, and fiber in their diets. As a result, obesity has reached epidemic proportions worldwide. Although obesity is not considered an eating disorder, it frequently co-occurs with such disorders. Common risk factors linking obesity and eating disorders include dieting, body dissatisfaction, weight/shape concerns, and unhealthy weight control behaviors. Both conditions have negative health consequences, which worsen when they occur together. In the Special Issue, the study by Pedram et al. [9] found that all-cause mortality of the lifetime self-reported history of eating disorders in the Canadian population was markedly elevated and considerably higher than that of other self-reported disorders, highlighting the seriousness of eating disorders and the urgent need for strategies that can help improve early diagnosis and a long-term plan for adequate interventions.

Finally, the study by Ivey et al. [10] analyzed the influence of the exposome, as a measure of all exposures of an individual in a lifetime, on the cardiometabolic risk profile. The study was carried out on a cohort of US Veterans and found evidence of structural relationships between diet, lifestyle, and demographic exposures and subsequent markers of cardiometabolic health. This study may provide valuable information toward the development of personalized interventions to improve cardiometabolic health outcomes based on the individual’s exposome. Additionally, it offers the potential to identify key determinants of cardiometabolic health and guide public health policies toward promoting healthy lifestyle choices.
